# A Real-Time Autonomous Dashboard for the Emergency Department: 5-Year Case Study

**DOI:** 10.2196/10666

**Published:** 2018-11-22

**Authors:** Junsang Yoo, Kwang Yul Jung, Taerim Kim, Taerim Lee, Sung Yeon Hwang, Hee Yoon, Tae Gun Shin, Min Seob Sim, Ik Joon Jo, Hansol Paeng, Jong Soo Choi, Won Chul Cha

**Affiliations:** 1 SAIHST Department of Digital Health Sungkyunkwan University Seoul Republic of Korea; 2 Department of Emergency Medicine Samsung Medical Center Sungkyunkwan University School of Medicine Seoul Republic of Korea; 3 Department of Emergency Medicine Chamjoeun Hospital Gwangju Republic of Korea; 4 Human Understanding Design Center (HUDC) Seoul Medical Center Seoul Republic of Korea; 5 Health Information Center Samsung Medical Center Seoul Republic of Korea; 6 Department of Digital Health Samsung Advanced Institute of Health Sciences and Technology Sungkyunkwan University Seoul Republic of Korea

**Keywords:** dashboard, development, emergency department, evaluation, health information technology, situation awareness, usability

## Abstract

**Background:**

The task of monitoring and managing the entire emergency department (ED) is becoming more important due to increasing pressure on the ED. Recently, dashboards have received the spotlight as health information technology to support these tasks.

**Objective:**

This study aimed to describe the development of a real-time autonomous dashboard for the ED and to evaluate perspectives of clinical staff on its usability.

**Methods:**

We developed a dashboard based on three principles—“anytime, anywhere, at a glance;” “minimal interruption to workflow;” and “protect patient privacy”—and 3 design features—“geographical layout,” “patient-level alert,” and “real-time summary data.” Items to evaluate the dashboard were selected based on the throughput factor of the conceptual model of ED crowding. Moreover, ED physicians and nurses were surveyed using the system usability scale (SUS) and situation awareness index as well as a questionnaire we created on the basis of the construct of the Situation Awareness Rating Technique.

**Results:**

The first version of the ED dashboard was successfully launched in 2013, and it has undergone 3 major revisions since then because of geographical changes in ED and modifications to improve usability. A total of 52 ED staff members participated in the survey. The average SUS score of the dashboard was 67.6 points, which indicates “OK-to-Good” usability. The participants also reported that the dashboard provided efficient “concentration support” (4.15 points), “complexity representation” (4.02 points), “variability representation” (3.96 points), “information quality” (3.94 points), and “familiarity” (3.94 points). However, the “division of attention” was rated at 2.25 points.

**Conclusions:**

We developed a real-time autonomous ED dashboard and successfully used it for 5 years with good evaluation from users.

## Introduction

An emergency department (ED) is a complex system designed to treat patients with various conditions simultaneously. Even though ED providers try to triage patients according to their clinical needs while managing scarce resources, these activities are often overwhelmed by the complexity and large volume of data. Emergency physicians are required to treat multiple patients while maintaining situational awareness of the ED surroundings [1]. This task is very challenging because it requires acquisition, processing, integration, and archiving of large data at multiple levels [2]. Thus, physicians frequently feel like they are losing control over the ED, which aggravates burnout and affects performance [3,4].

Moreover, many EDs are already overcrowded, thus increasing the complexity [5-7]. While ED overcrowding remains a major health care issue, it significantly and adversely affects quality of care by influencing major quality factors, such as timeliness, effectiveness, efficiency, safety, and patient-centeredness, resulting in increased mortality and morbidity [5,8-10]. Multiple studies have suggested the use of system engineering and science to improve ED performance, streamline the process, and improve the throughput [11-13]. However, a strategic approach to identify process delays and supply-demand mismatch using traditional hospital information systems (HISs) is not feasible. It is important to monitor and manage the ED as a whole.

The dashboard is “a visual display of the most important information needed to achieve one or more objectives” [14]. Because the situation in the ED affects the quality of care and patient outcomes, clinicians use different diagnostic and treatment strategies depending on the situation [15]. Therefore, recognizing the correct situation is becoming increasingly important to emergency medical clinicians [16].

While a quality dashboard helps decision making at the organization level, a clinical dashboard helps decision making regarding patient care [17]. A dashboard that fits the changing situations in the ED in the real time must have the characteristics of both quality and clinical dashboards [17]. Recent studies on the ED dashboard system have reported its potential to improve patient safety, situation awareness, and workflow [15,18], but such studies have not addressed long-term experiences and association between the introduction of the dashboard and mental workload of the users. In particular, ensuring real-time availability is challenging and important because nonreal–time dashboards cannot support decision making [15].

This study aimed to describe the development of a real-time organizational dashboard for the ED and to evaluate its usability.

## Methods

This study was approved by the Institutional Review Board of the study site (Institutional Review Board File #SMC 2018-01-040-001).

### Study Setting

The study was carried out in a metropolis: Seoul. It was undertaken at an ED with an annual visit volume of 79,000 patients in a tertiary teaching hospital. The hospital has about 2000 inpatient beds. This ED is one of the most overcrowded EDs in the country [19].

The HIS was in use in this ED since 1994, supported by electronic medical records and a picture archiving and communication system. Although the history of this HIS is long and its technical quality was one of the most advanced in the country, no ED-specific dashboard system was ever used in this hospital.

### Development

#### The Happinovation and the Happy Emergency Room Team

In 2012, an institution-wide project, the “Happinovation” (Happy innovation), was initiated to enhance patient and provider happiness through process and hardware innovations. The “Happy Emergency Room Team” was formed as a satellite team for the overall project. The team focused on visualizing the ED process for providers and patients.

Specifically, 2 subprojects were implemented. One was a visualization project for providers—an electronic dashboard to develop visualization of ED performance status on wall-mounted monitors and PCs. The other was a visualization project for patients and their families—a wall-mounted electronic dashboard, kiosks, and tablets.

The multidisciplinary Happy Emergency Room Team included 6 physicians, 4 nurses, 1 administrator, 2 quality improvement team members, 2 consultants, and 2 designers. While hospital staff provided inputs, consultants and designers tried to see things from patients’ perspective, thus balancing the conclusion. Several rounds of discussions and debates took place before the first dashboard principle and design feature was created.

#### Dashboard Principle

The team had agreed on the following 3 dashboard principles that define the characteristics of the dashboard.

##### Anytime, Anywhere, at a Glance

The dashboard was designed to be like a traditional whiteboard for the ED, which means that it should be mounted on a wall and be visible to providers from about 2-5 m away. It should be in a static mode, without flipping the screen, such that providers would not waste any time to find the desired information [20]. Since multiple providers should be able to access the information, interactivity was not feasible. We did not include a writing and communication function from the dashboard because it was designed to only offer providers the overall ED status at a glance [15,21].

##### Minimal Interruption to Workflow

The dashboard should be integrated with the clinical workflow. An unfavorable clinical workflow often changes after implementing health information technology [21-23]. However, the Happy Emergency Room Team worried that the dashboard could be a source of botheration. Therefore, we designed it such that it would not require any additional inputs. This means that all data should come from the legacy system.

**Figure 1:**
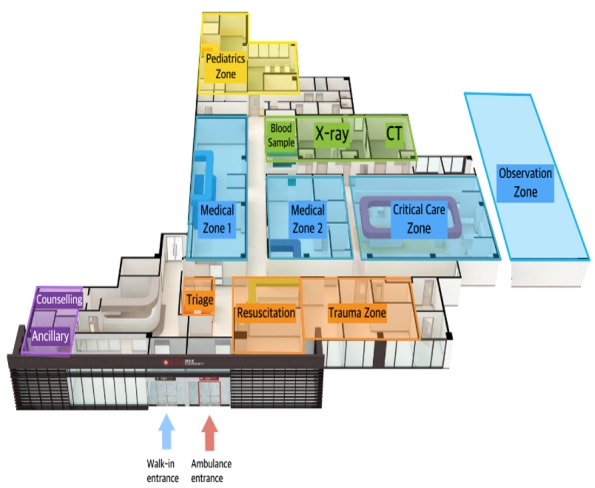
Geographical layout of the emergency department.

All information on the dashboard is automatically generated from previous columns of HIS. For example, the moment at which a physician’s order or barcode of a clinical sample is input by a nurse, it is linked to the dashboard system such that the whole operating process could be automatically marked and shown without additional inputting processes. This is also different from traditional dry-erase whiteboards [24].

##### Protect Patient Privacy

It is important that patients’ privacy be secured [25]. Because the screen is physically accessible to patients and families, most information should be deidentified and symbolized to prevent unnecessary misunderstandings and debates.

#### Design Features

The team adopted the following 3 design features for the dashboard:

##### Geographical Layout

The dashboard should indicate the ED floor plan. A geographical layout would give intuitive information to ED providers on what is going on where in the ED. It is also very common that ED providers recognize and communicate about patients with their bed locations and not with their numbers or names, which made it more intuitive and effective to use a geographical layout rather than a patient list (Figure 1).

##### Patient-Level Alert

All beds and chairs were symbolized to stand for a patient to provide patient-specific information to ED clinicians. Additional patient-level information was included to provide a real-time alert to providers through encoded colors and symbols, especially regarding process delays. The main objective of this concept was to provide ED staff with patient-level alerts such that a provider could immediately notice delays in catering to patients.

##### Real-Time Summary Data

The dashboard would show summary statistics regarding ED performance in the real time. While in-depth information for each patient is available in the pre-existing HIS, a real-time summary depends mostly on subjective feeling of individual providers, which varies significantly due to the lack of information on summary information over ED state. With summary data, providers would be able to reschedule clinical processes for their patients’ efficient journey. For example, a nurse could direct a patient to the X-ray if there is a long queue for a co-ordered computed tomography.

#### Prototyping

Initially, multiple measures were suggested for the dashboard. Measures were chosen and categorized based on a “conceptual model of ED crowding.” The model consisted of 3 factors, among which the throughput factors, which reflect the internal process of ED care, were mostly demonstrated with the dashboard [13]. Among input factors, which are components that contribute to the demand for ED services, patient severity and measure of visits were included. Cautions on infection information were also included since they were well correlated with severity and patient allocation within the ED and hospital. Throughput factors were divided into 2 sections: structures and functions. Output factors, such as boarding and discharge data, were also included. Boarding pertains to the inability to admit a patient to a ward due to the lack of inpatient beds even through the patient is determined to be admitted.

**Figure 2:**
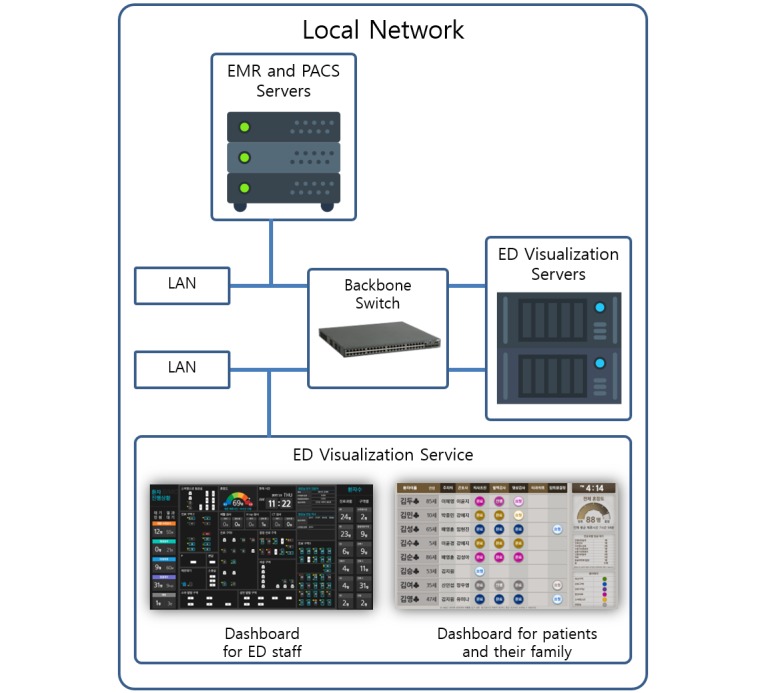
Transfer of electronic medical record (EMR) and picture archiving and communication system (PACS) data to the emergency department (ED) visual architecture platform. LAN: Local Area Network.

We used a Windows server as the ED visualization platform to support various devices in the ED, including the dashboard and kiosks for patients and mobile devices. Our entire platform was developed and deployed on 2 Windows 2008 servers, each with a 1200-GB hard drive and 20-GB memory, and 2 4-core Intel Xeon 2.4 GHz processors. The servers queried the measurements mentioned above from the electronic medical records and picture archiving and communication system servers. A Windows communication foundation was used as a visualization tool (Figure 2).

#### Evolution of the Emergency Department Dashboard

We developed and updated the dashboard over 5 years. During the observation, the ED had gone through a Middle East Respiratory Syndrome outbreak, followed by a major structural and functional renovation [26]. The ED dashboard adopted such changes and evolved through the process.

### Evaluation

#### Selection of Participants

Inclusion criteria were those currently working as ED physicians and triage or charge nurses and those using the dashboard. Participants were recruited from January 1 to February 10, 2018.

#### Intervention

Participants responded to 20 questions on a 5-point Likert scale after completing a consent form. The first 10 items of the questionnaire were derived from the system usability scale (SUS) to investigate the usability of the dashboard [27]. The last 10 items were derived from the situation awareness index (SAI), which we composed based on the Situation Awareness Rating Technique (SART) [28]. The SAI aimed to assess whether ED physicians and nurses were using the dashboard to help them recognize the situation. The SAI includes the constructs of the SART as a whole, and small modifications were made to adjust it to fit the dashboard.

After completing the questionnaire, the participants received about US $8 as compensation for participating in the survey.

#### Outcome Analysis

The SUS scores were interpreted using an adjective rating scale [29].

The SAI score was calculated using the following formula:

SAI = {Q11 + Q12 + Q13 + Q14 + Q15 + Q16 + (6 - Q17) + Q18 + Q19 + Q20} / 10, where Q: question number.

The SAI scores were also examined using descriptive analyses.

## Results

### Design and Structure

#### Introduction to the First Version of the Emergency Department Dashboard

The semicircle-shaped indicator in the upper central area represents the complexity of the ED and presents the expected mean length of stay of a current patient in the ED. The semicircle borrows the scheme of the traffic light so that the user intuitively grasps the current situation of the ED.

A semitransparent colored square represents each section of the ED and matches the geographical layout presented in Figure 1. The squares are not visible on the actual dashboard. Small icons, such as that shown in the red circle, indicate patients, and they intuitively inform the user about the patient’s journey through the color corresponding to the patient’s process on the left.

**Figure 3:**
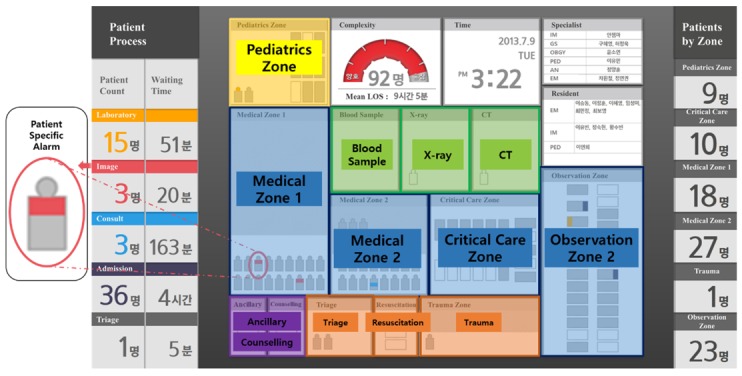
The first version of the emergency department dashboard.

The left side of the dashboard presents a summary of the patient process. This information allows the physician to set up a patient diagnosis strategy and the nurse to determine the order of various tests. The central area of the dashboard reflects the geographical layout of the ED. Here, individual patients’ specific information is displayed, such as real-time clinical processes. The right side of the dashboard presents the number of patients by zone, thereby enabling efficient distribution of medical labor (Figure 3).

#### Evolution of the Emergency Department Dashboard

**Figure 4:**
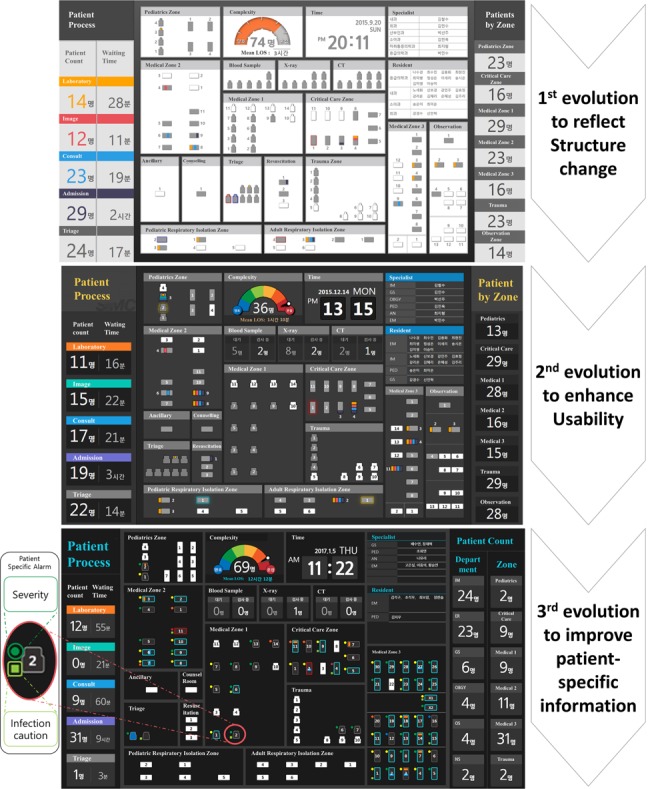
Evolution of the emergency department dashboard, showing the second, third, and final versions.

Major difference between versions 1 and 2 of the dashboard is that the latter reflects a geographical change. In 2015, the ED of the study site underwent a Middle East Respiratory Syndrome outbreak, and a respiratory isolation area was established and operated. This structural change to the ED was reflected on the dashboard (Figure 4).

In version 3, a revision was made to improve usability. We updated the dashboard to change the overall color coding. By doing so, we were able to reflect the users’ suggestions, such as “difficulty to identify bed status” (Figure 4).

A major change in version 4 is the improvement of patient-specific information.

The circle next to the patient indicates the mapping of the Korean Triage and Acuity Scale score from 1 to 5, presented from red to green. Additionally, the rectangle next to the patient reflects “infection caution,” such as “air caution” and “blood caution” (Figure 4).

### Evaluation

#### Participant Characteristics

A total of 52 participants were recruited; 25 were physicians and 27 were nurses. In the physician group, 17 were males and 8 were females; in the nurse group, only 2 were males and 25 were females. The participants’ years of experience also varied; 8 had worked for less than 3 years, 10 had worked for 3-5 years, 19 had worked for 5-10 years, and 15 had worked for over 10 years (Table 1).

#### System Usability Scale

The SUS score of the ED dashboard system was 67.6 points (Table 2). The SUS score showed a slight difference between the 2 groups; the physician and nurse groups had scores of 67.5 and 67.69 points, respectively. We could interpret our result as follows: acceptability of the dashboard was “marginally high,” and the adjective rating was “OK-to-Good” [29]. These findings indicate that the participants used it very frequently. They also felt that this system was easy to learn and use.

#### Situation Awareness Index

The overall SAI score was 3.87 points, and the score of the physician group (3.95) was higher than that of the nurse group (3.80). The top 5 rated items were “concentration support” (4.15), “complexity representation” (4.02 points), “variability representation” (3.96 points), “information quality provided” (3.94 points), and “familiarity of dashboard” (3.94 points). However, the score for “division of attention” was 2.25 points (Table 3).

**Table 1:** Characteristics of participants.

Characteristic		Physician (n=25)	Nurse (n=27)	Total (N=52)
**Age group, n (%)**				
	20s	2 (8)	6 (22)	8 (15)
	30s	19 (76)	19 (70)	38 (73)
	40s	1 (4)	2 (7)	3 (6)
	50s	3 (12)	0 (0)	3 (6)
**Sex, n (%)**				
	Male	17 (68)	2 (7)	19 (37)
	Female	8 (32)	25 (93)	33 (64)
**Years of experience, n (%)**				
	0-3	8 (32)	0 (0)	8 (15)
	3-5	6 (24)	4 (15)	10 (19)
	5-10	4 (16)	15 (56)	19 (37)
	>10	7 (28)	8 (30)	15 (29)

**Table 2:** System usability scale scores.

Item	Physician, mean (SD)	Nurse, mean (SD)	Total, mean (SD)
Q1. I think that I would like to use this dashboard frequently.	4.52 (0.7)	4.15 (0.5)	4.33 (0.6)
Q2. I found the dashboard unnecessarily complex.	2.44 (0.9)	2.52 (1.0)	2.48 (0.9)
Q3. I thought the dashboard was easy to use.	3.76 (0.7)	3.85 (0.6)	3.81 (0.7)
Q4. I think that I would need the support of a technician to be able to use this dashboard.	3.08 (1.1)	2.89 (1.1)	2.98 (1.1)
Q5. I found that the various functions in this dashboard were well integrated.	3.56 (0.8)	3.78 (0.7)	3.67 (0.7)
Q6. I thought there was too much inconsistency in this dashboard.	2.24 (0.9)	2.07 (0.7)	2.15 (0.8)
Q7. I would imagine that most people would learn to use this dashboard very quickly.	3.72 (0.9)	3.93 (0.6)	3.83 (0.8)
Q8. I found the dashboard very cumbersome to use.	2.12 (0.7)	2.11 (0.6)	2.12 (0.7)
Q9. I felt very confident using the dashboard.	3.84 (0.9)	3.67 (0.8)	3.75 (0.9)
Q10. I needed to learn a lot of things before I could get going with this dashboard.	2.52 (1.1)	2.70 (1.1)	2.62 (1.1)
System usability scale score	67.50 (12.0)	67.69 (11.0)	67.60 (11.4)

**Table 3:** Situation awareness and dashboard results.

Construct	Item	Physician, mean (SD)	Nurse, mean (SD)	Total, mean (SD)
Instability representation	Q11. The dashboard adequately represents the instability of the ED^a^.	3.96 (1.0)	3.78 (0.7)	3.87 (0.8)
Complexity representation	Q12. The dashboard adequately represents the complexity of the ED.	4.12 (0.9)	3.93 (0.6)	4.02 (0.8)
Variability representation	Q13. The dashboard contains key elements that are changing in the ED.	4.08 (0.6)	3.85 (0.8)	3.96 (0.7)
Arousal support	Q14. The dashboard helps me be alert and clearer.	3.92 (0.9)	3.74 (0.7)	3.83 (0.8)
Concentration support	Q15. The dashboard helps me focus on the situation in the ED.	4.20 (0.6)	4.11 (0.6)	4.15 (0.6)
Spare mental capacity support	Q16. I can acquire additional mental capacity in a pressing ED situation.	3.60 (1.2)	3.48 (0.9)	3.54 (1.0)
Division of attention	Q17. The dashboard distracts attention from important tasks of the ED.	2.12 (0.7)	2.37 (0.9)	2.25 (0.8)
Information quantity provided	Q18. The quantity of information provided by the dashboard is appropriate for performing ED tasks.	3.84 (0.9)	3.67 (0.8)	3.75 (0.8)
Information quality provided	Q19. The quality of information provided by the dashboard is appropriate for performing ED tasks.	3.92 (1.0)	3.96 (0.6)	3.94 (0.8)
Familiarity of dashboard	Q20. I can perform ED tasks more proficiently using the dashboard.	4.00 (0.9)	3.89 (0.8)	3.94 (0.9)
Situation awareness index	—	3.95 (0.6)	3.80 (0.5)	3.87 (0.6)

^a^ED: emergency department.

## Discussion

### Principal Findings

The ED dashboard was successfully developed and implemented. The system is independent of manual input and is fully connected to the legacy HIS. The graphical and statistical concepts were determined during the developmental period, and they were upgraded gradually. Though clinical dashboards for EDs have been developed in other studies [15,30-32], this study is the first to examine its long-term use and conduct serial upgrades.

We used SUS, a formal, highly validated usability test, and obtained a score of 67.6 points from physicians and nurses. This score could be interpreted as indicating “marginally high acceptability” with “OK-to-Good usability.” Additionally, the ED staff responded that the dashboard presented the situation in the ED effectively and that they could better focus on the changing situation in the ED by using it. The quality of the information provided by the dashboard was rated high; however, the quantity of information was rated relatively low. There is a need for a systematic investigation to establish the information that ED staff seek and a subsequent improvement plan to reflect the same in the system.

### Clinical Aspects

It has been one of the major responsibilities of ED staff to keep patients’ processes on track and ensure timely results [33]. To do so, ED providers had to call numerous departments and browse through multiple windows repeatedly during their duty time. As the tasks and volume of ED work-ups grew, this timekeeping function had become significantly heavy. The ED dashboard described in this study focused on providing a visual representation of this “hidden” information pertaining to the ED process, such that ED providers could plan and carry out their tasks proactively.

The visualized information not only pertained to individual patients’ processes but also reported the department’s performance status. As the government and insurance companies focus on performance as a group of patients, it has become an essential job for the system administrator to be able to assess real-time statistics. ED providers’ responsibility as administrators demands tools like our dashboard during their work.

Our ED dashboard affects the workflow of various medical personnel in the ED in a variety of ways, ranging from simple information delivery to clinical decision-making support. If the dashboard indicates that the ED is extremely overcrowded, the ED chief could contact the national acute care system to control the transfer of new patients. Additionally, physicians could grasp the timely information of each patient using this dashboard. The charge nurse could improve the efficacy of the ED by reassigning each nurse to more appropriate zones based on the information obtained from the dashboard. Triage nurses could also use the dashboard to allocate patients to appropriate treatment zones.

### Comparison With Previous Work

Recent review articles have indicated that an ED dashboard could be useful for “saving time and reducing the risk of errors or delay” [18]. However, the association between the introduction of an electronic dashboard and the mental workload of ED staff is controversial. Further, there is little evidence to support that it improves clinical outcomes [18].

A major difference between our system and other electronic boards is that our dashboard does not allow manual or direct input of patient-specific information. We have fully synchronized our dashboard with the legacy system such that physicians’ and nurses’ additional inputs are not required to use the dashboard. By achieving this goal, our system was found to provide effective arousal support, concentration support, and spare mental capacity (3.83 points, 4.15 points, and 3.54 points, respectively) even in the busy clinical setting.

### Limitations

First, this is a single-center case study with its unique HIS. Its feasibility and usability should be validated in other institutions. Considering that the implementation of this system in other institutions has been discussed, subsequent investigation on ED dashboard utilization is expected in the near future.

The measures used for the dashboard are not universally agreed upon. They have mainly been used in a highly crowded EDs of a teaching hospital. When used in smaller EDs, the measures of interest would be different. Additionally, the measures were not compared with the national standard, which requires further research.

The SAI has not been validated. We searched for questionnaires to investigate the association between dashboards and situation awareness, but we could not find any that suited our purpose. Therefore, we developed a questionnaire based on the SART and applied it in this study. However, this questionnaire is not validated, and therefore, its interpretation value is limited.

Finally, we could not investigate the association between the effectiveness of the ED dashboard and clinical outcomes. However, it is difficult to identify this association because the ED is one of the most complex systems affected by several uncontrollable factors. Therefore, multicenter comparative studies need to be conducted to examine this association.

### Conclusions

We developed a real-time autonomous ED dashboard and successfully used it for 5 years. ED physicians and nurses rated the usability of the ED dashboard as “OK-to-Good.” We realize that continuous maintenance is important because the dashboard should reflect the situation of the ED.

## Abbreviations

ED: emergency department
HIS: hospital information system
LAN: Local Area Network
SAI: situation awareness index
SART: Situation Awareness Rating Technique
SUS: system usability scale
